# Supplementary material for: Content-rich biological network constructed by mining PubMed abstracts
Source: BMC Bioinformatics. 2004 Oct 8;5:147. doi: 10.1186/1471-2105-5-147 (PMC528731; doi:10.1186/1471-2105-5-147)
Supplement: Additional File 5 — The original Chilibot query results of the term "long-term potentiation (LTP)" and 22 other terms, limiting the latest references analyzed to the years 1990, 1995, 2000, and 2004. [file 1471-2105-5-147-S5.bz2 › chilibotAdditionalFile5/ltp2004/html/TRKA.html]

 


**TRKA** (Input: TRKA ) 

---


**Google Searches:** Entire Web  | EDU domain only  | PDF files only

.

**External Links:** OMIM |
LocusLink
| Swissprot
| GeneCards

  
**Maps of TRKA**

Simple
Complete
graph in
radiant
tree
square
layout.

**New Hypothesis !**

**Synonyms** 

- trk a   [PubMed]

- trka   [PubMed]

**Synopsis**

- These results suggest that the NGF effect is mediated by the high affinity NGF receptor, Trk A  [**TRKA**] .  J Biol Chem, 1997 
  [23]

- These observations suggest that **TrkA** can activate different survival signalling pathways, which can interfere with specific apoptotic pathways.  Oncogene, 1998 
  [19]

- This distribution pattern of Trk A  [**TRKA**]  suggests that its ligand, NGF, exerts significant trophic effects in the rodent inner ear.  Hear Res, 2004 
  [19]

- Moreover, recruitment of PLC gamma to **TrkA** is essential for NGF mediated potentiation of channel activity, and biochemical studies suggest that VR1 associates with this complex.  Nature, 2001 
  [19]

- TrkB and **TrkA** like immunoreactivity was never found.  Anat Embryol (Berl)Anat Embryol (Berl), 2001 
  [18]

- We show here that their respective receptors, **TrkA**, TrkB, TrkC, as well as p75 NTR the low affinity NGF receptor, are expressed in vitro by the nasal ensheathing cells.  Glia, 2004 
  [16]

- Cells from the immune system have been detected in CNS injury and found to produce and secrete a variety of neurotrophins such as NGF, BDNF, NT 3 and NT 4 5, and to express similarly to neuronal cells, members of the tyrosine kinase Trk receptor family such as **TrkA**, TrkB and TrkC.  Prog Brain Res, 2004 
  [16]

- K252a 10 100 nm, BAPTA AM 25 microm, and Cd 2 200 microm prevented NGF enhancement of ACh release, suggesting the involvement of **TrkA** receptors, calcium, and voltage gated calcium channels, respectively.  J Neurosci, 2001 
  [16]

- Mechanistically, NGF stimulation of PC12 cells resulted in a weak or possibly indirect association between **trkA** and PI 3 kinase.  J Neurosci Res, 1995 
  [14]

- Furthermore, even after transfection with exogenous TRK A  [**TRKA**] , early responses were restored but later events such as neurite outgrowth did NOT occur, suggesting that downstream responsiveness is blocked as well.  Prog Clin Biol Res, 1994 
  [12]

- These results indicate that in developing cortical neurons, activation of AMPA KA receptors by KA may induce expression, followed by activation of **TrkA** via PLC signaling and intracellular calcium elevation and hence increase reception of NGF on KA challenged neurons.  J Neurochem, 2000 
  [11]

- Furthermore, TrkC14 and TrkC25 have only a low intrinsic tyrosine kinase activity, and insertion of the TrkC14 kinase insert into **TrkA** at an equivalent position results in a dramatic reduction of the kinase activity and signaling capabilities of **TrkA**.  J Biol Chem, 1996 
  [11]

- Uniform exposure of **TrkA** expressing neurons to NGF resulted in a cross desensitization of turning responses induced by a gradient of netrin 1, brain derived neurotrophic factor BDNF, or myelin associated glycoprotein MAG  Neuron, 1999 
  [11]

- These results clearly implicate c Crk II in the NGF signaling pathway and support the concept that more than one signaling molecule known to participate in the activation of Ras associates with **TrkA** upon NGF treatment.  Oncogene, 1996 
  [11]

- NGF does NOT result in **TrkA** phosphorylation.  Brain Res Dev Brain Res, 1996 
  [11]
